# 

**Published:** 1895-06

**Authors:** 


### CONTENTS FOR JUNE, 1895.

ORIGINAL COMMUNICATIONS. PAGE.

The Etiology, Diagnosis and Treatment of Diphtheria. By W. L. CONKLIN, M. D....... 641
Antitoxin in the Treatment of Diphtheria. By CHARLES D. AARON, M. D................ 656
Enteroclysis in the Various Forms of Enterocolitis and the Zymotics Generally. By F. W. BARTLETT, M. D....... ........................................................ 663
Note on Smoked Glasses. By RICHARD H. SATTERLEE, M. D.............................. 666
State Commissioners in Lunacy. By GROSVENOR R. TROWBRIDGE, M. D................ 666

PROGRESS IN MEDICAL SCIENCE.

State Medicine, Public Health, Hygiene and Bacteriology.............................. 668

STATE MEDICAL EXAMINATIONS.

Amendments to the Medical Practice Law..... .... ........................................ 675

EDITORIAL.

College Commencements and Alumni Association Meetings .............................. 678
Topics of the Month................. ......... ................................................ 686

Obituary ......................................................................................... 691
Personal ......................................................................................... 692
Society Meetings................................................................................ 693
Hospital Notes.................................................................................. 694
Medical College Notes........................................................................ 694
Book Reviews.................................................................................... 695
Literary........................................................................................... 703

